# Integrative Management of Bone Deformities in Osteogenesis Imperfecta: A Case Report on Zoledronic Acid and Corrective Osteotomy

**DOI:** 10.7759/cureus.70880

**Published:** 2024-10-05

**Authors:** SreeHarsha Damam, Amar Taksande, Revat J Meshram

**Affiliations:** 1 Department of Pediatrics, Jawaharlal Nehru Medical College, Datta Meghe Institute of Higher Education & Research, Wardha, IND

**Keywords:** bisphosphonate, corrective osteotomy, osteogenesis imperfecta, pediatric orthopedics, zoledronic acid

## Abstract

Osteogenesis imperfecta (OI) is a hereditary genetic condition characterized by brittle bones that are easily broken. Surgical intervention is necessary frequently to treat fractures and deformities in patients diagnosed with OI. In this case, we discuss a case of a nine-year-old male child diagnosed with OI. This boy was previously operated on one year back for a femur fracture with osteotomy and internal fixation with nails, followed by implant removal at a later stage almost one year later. This boy was brought to our hospital with convex deformities of femur and tibia. Upon evaluation and review of this boy’s case, the decision was made to administer a zoledronic acid infusion cycle over three days to enhance bone strength. Following this pharmacological therapy, this patient was planned for corrective osteotomy and internal fixation of the femur. This case underscores the complexities of managing OI and also highlights the importance of the therapeutic role of bisphosphonates like zoledronic acid alongside surgical intervention to address these kinds of bony deformities and improve patient outcomes.

## Introduction

Osteogenesis imperfecta (OI) is a hereditary disorder characterized by brittle bones that are prone to fractures even with little or no trauma. This genetic condition is the result of mutations in the genes named COL1A1 and COL1A2, which lead to defects in collagen synthesis [1]. The prevalence of this condition ranges from one in 15,000 to 20,000 births [2]. OI clinically manifests in a variety of ways, ranging from mild cases with minimal or no fractures to severe forms that result in significant fractures and skeletal deformities [3]. The severity of OI varies from mild to severe forms; mild may go undetected until maturity to severe forms that can be fatal at birth [4]. Management of OI includes a multidisciplinary approach, which incorporates medical therapies such as bisphosphonates, physical rehabilitation, and also surgical interventions to treat fractures and bony deformities [2]. Recent advancements in the understanding of genetics have significantly improved the outcomes in these patients [5].

## Case presentation

**Figure 1:**
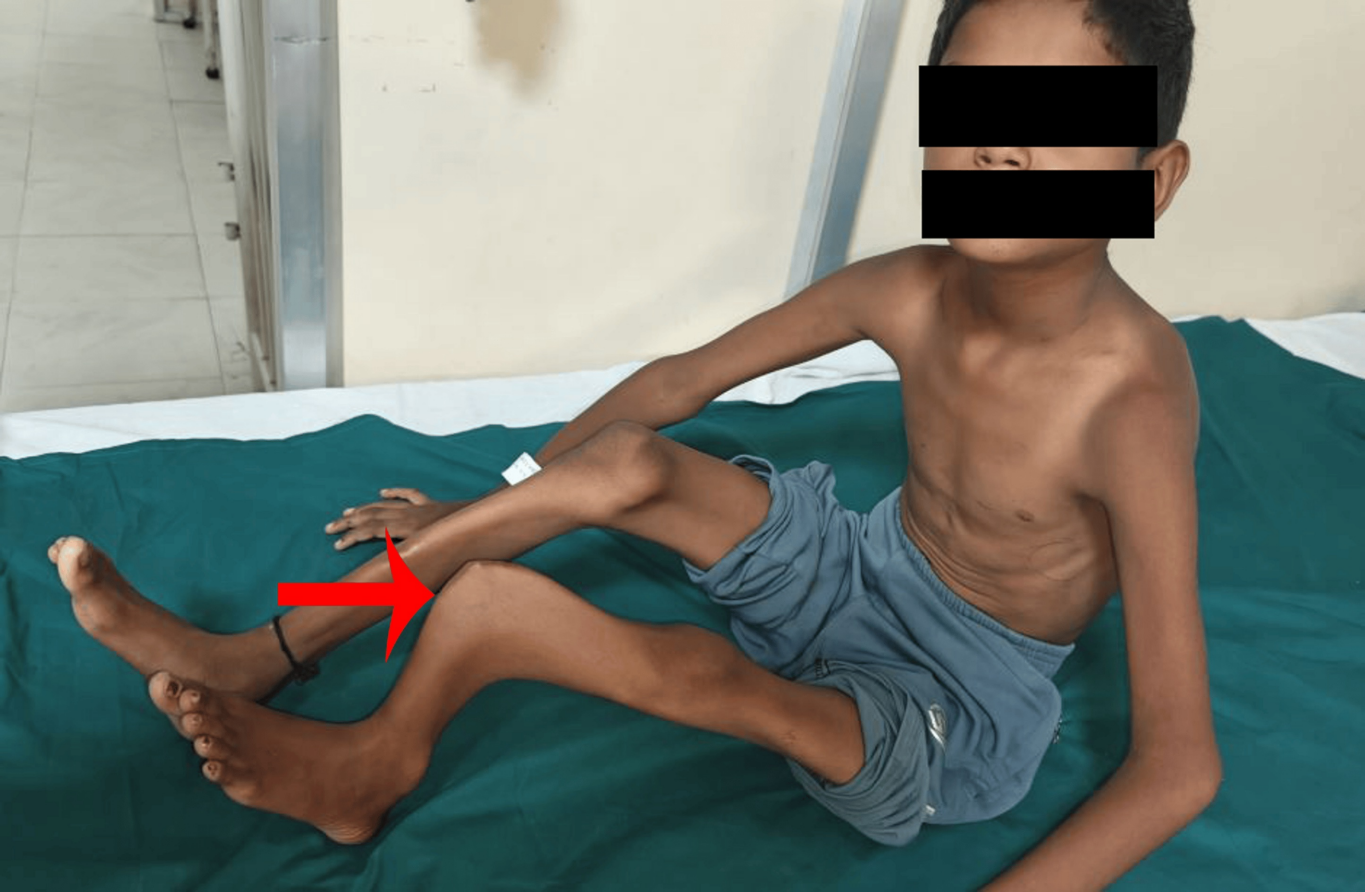
Lateral view of the left leg showing pronounced anterior bowing of the tibia.

**Figure 2:**
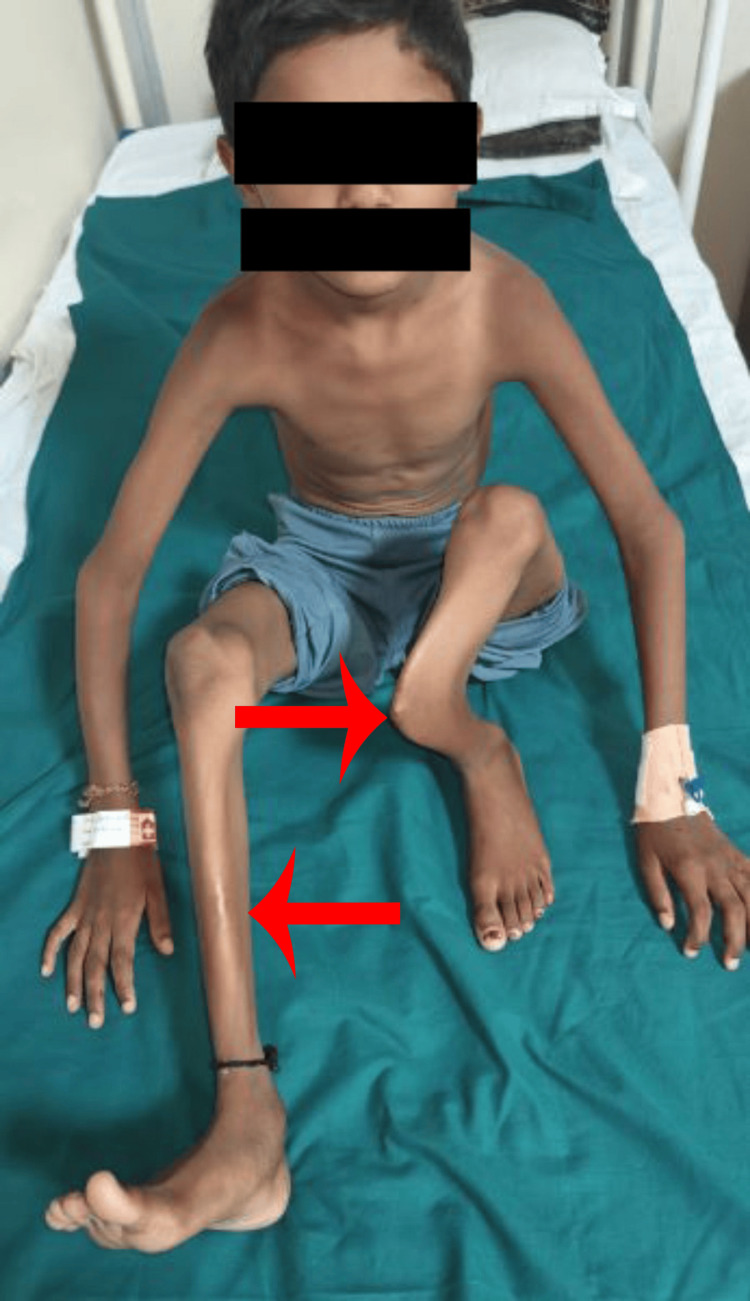
Anterior view of both lower limbs demonstrating bilateral bowing, with a more significant deformity in the left leg.

A nine-year-old boy was brought with a known diagnosis of OI with chief complaints of progressive deformities of both lower limbs over the past eight years with a history of recurrent fractures and skeletal deformities. As narrated by the mother, the child was initially asymptomatic till years of age when the child started developing bilateral bowing of legs of insidious onset, predominantly affecting the left limb. Later, the child had difficulty walking with no associated history of trauma, pain, or swelling at the site or deformity. At six years of age, he had a left femur fracture for which a surgical intervention was performed, although the documents are not fully available. This surgery involved an implant placement and was removed approximately a year later due to reasons unknown. There is no other noteworthy past medical or surgical history. On inspection, skin over the affected limbs appeared normal with healed scar marks over the lateral aspect of the left femur. Anterior bowing of the left tibia, mild bowing of the right leg, and wasting over thigh and calf muscles were evident (Figures 1, 2). No local rise in temperature or bony tenderness over the left femur and tibia was noted. Active movements of the knee, ankle, and toes were intact with palpable pulses and normal sensations distally. The laboratory investigations were done (Table 1). Bone health was optimized by monitoring and maintaining appropriate vitamin D levels. The observed value of 47.1 ng/ml falls within the sufficient range (30-100 ng/ml), ensuring adequate support for bone mineralization and overall skeletal health.

**Table 1:** This table summarizes the laboratory investigations conducted on the patient.

Parameter	Reference range	Observed value
Platelet count	150,000-450,000/cumm	225,000/cumm
Total leukocyte count	4,000-11,000/cumm	5,900/cumm
Hemoglobin	13-15 g/dl	12.4 g/dl
Thyroxine	5.53-11 μg/dl	5.37 μg/dl
Triiodothyronine	0.97-1.69 ng/ml	1.38 ng/ml
Thyroid-stimulating hormone	0.46-4.68 μIU/ml	2 μIU/ml
Conjugated bilirubin	0-1.1 mg/dl	0.2 mg/dl
Unconjugated bilirubin	0-0.3 mg/dl	0.2 mg/dl
Total bilirubin	0.2-1.3 mg/dl	0.4 mg/dl
Globulin	2.3-3.5 mg/dl	3.3 mg/dl
Total protein	6.3-8.2 g/dl	7.5 g/dl
Albumin	3.5-5 g/dl	4.2 g/dl
Creatinine	0.6-1.2 mg/dl	0.36 mg/dl
Urea	9-20 mg/dl	20 mg/dl
Alanine transaminase	<50 U/l	41 U/l
Aspartate transaminase	17-59 U/l	35 U/l
Alkaline phosphatase	38-126 U/l	110 U/l
Calcium	8.4-10.4 mg/dl	9.1 mg/dl
Vitamin D	Deficient: <20 ng/ml	47.1 ng/ml
Potassium	3.5-5.1 mmol/l	4.1 mmol/l
Sodium	136-145 mmol/l	136 mmol/l
Phosphate	2.5-4.5 mg/dl	3.5 mg/dl
Erythrocyte sedimentation rate	2-13 mm/h	4 mm/h

**Figure 3:**
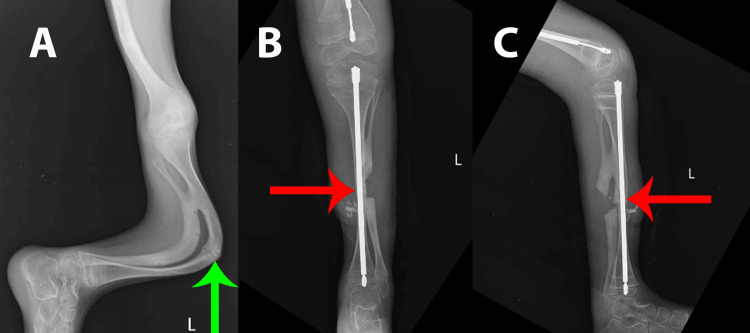
(A) Preoperative lateral X-ray of the left tibia with a significant bowing deformity, particularly at the distal third, with a sharp curvature (indicated by the green arrow). (B) Immediate postoperative X-ray with the placement of an intramedullary rod within the left tibia (indicated by red arrow). The alignment of the bone has been corrected, with the intramedullary rod stabilizing the tibia to prevent further deformity. The osteotomy site is visible, and the bone segments are aligned. (C) Another immediate postoperative X-ray from a lateral view with the intramedullary rod in place along the tibia. This view provides additional confirmation of the stability achieved postoperatively.

**Figure 4:**
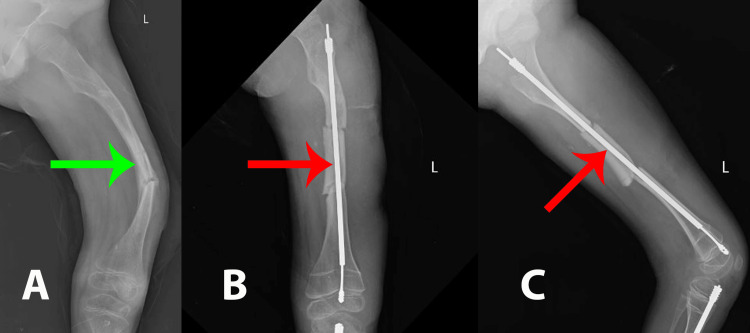
(A) Preoperative X-ray with severe bowing of the left femur with significant curvature (indicated by the green arrow), a characteristic deformity seen in osteogenesis imperfecta. The bone appears thin and fragile, with a notable varus deformity. (B) Immediate postoperative X-ray demonstrating the placement of an intramedullary rod in the left femur indicated by the red arrow. There is also evidence of osteotomy, with proper alignment of the femoral segments. (C) Another immediate postoperative lateral view X-ray with the intramedullary rod in place along the left femur. The red arrow indicates the position of the rod, confirming the corrected alignment from a different perspective. This lateral view highlights the successful stabilization and positioning of the rod, further ensuring the bone's structural support post surgery.

Intravenous zoledronic acid was part of the patient's treatment plan. In particular, a daily dose of 1 mg/kg was given, diluted in 500 ml of normal saline, and infused over the course of eight hours on three consecutive days prior to surgery. The optimization of bone health, including monitoring and adjusting vitamin D levels, was completed two days prior to surgery to ensure appropriate levels before the procedure. The patient had preoperative treatment followed by surgery. The clinical image of the patient demonstrating the bilateral bowing and deformities of the lower limbs is provided below, along with the preoperative (Figures 3A, 4A) and postoperative X-ray images (Figures 3B, 3C, 4B, 4C) showing the extent of deformity and the correction achieved after surgical intervention. Under general anesthesia, soft tissue reconstructions were done for deformities of both the left femur and tibia. For the tibia, osteotomy was done at the mid-shaft using a saw to ensure precision and minimize trauma to the surrounding tissues, removing a 3 cm bony part after superficial dissection and periosteal elevation. Using intraoperative fluoroscopy, this alignment was verified and was secured by Fassier-Duval (FD) rod insertion through a patellar tendon split incision (Figures 3B, 3C). Similarly at the femur, osteotomies were performed at the proximal and distal regions and the alignment was verified and secured through an intramedullary FD rod (Figures 4B, 4C). Postoperatively, the patient exhibited stable vital signs, intact distal circulation, and also adherence to non-weight-bearing protocols. Weight-bearing was initiated based on both the time frame and the healing observed on the X-ray. Initially, a gradual weight-bearing approach was implemented, with monitoring for any signs of complications. X-ray assessments were conducted to confirm adequate healing before progressing to full weight-bearing.

Following successful surgical correction, the patient was advised to continue oral medications and maintain the leg cast as prescribed for a duration of six weeks to provide adequate support during the initial healing phase. Physical therapy was initiated shortly after surgery to optimize postoperative mobility and functionality. The initial phase of physical therapy focused on gentle exercises that could be performed without weight-bearing on the affected leg. This included ankle pumps to encourage circulation and prevent stiffness and quad sets to engage the thigh muscles without placing stress on the knee joint. The patient was also advised to avoid contact with moisture, refrain from inserting objects into the cast, and avoid modifying it. Vigilance is crucial for detecting signs of complications, such as blackening of the toes, swelling, or unusual numbness in the left leg, which would prompt immediate consultation. Additionally, bisphosphonates were avoided for three months postoperatively to allow for proper healing and minimize potential complications. After the removal of the cast, no bracing was used, as the physical therapy regimen was deemed sufficient for recovery. Stationary cycling was also encouraged as soon as the cast was off, providing a low-impact option for improving cardiovascular fitness. Throughout the process, the patient was provided with a home exercise program to maintain strength and mobility, with regular follow-ups to monitor progress and adapt the regimen as necessary.

## Discussion

Due to the genetic basis and the varied presentation of bone fragility and deformities, this poses significant challenges in the management of OI. This case of a nine-year-old male child highlights the complexity of treating OI and the integrative approach required for optimal patient outcomes. These surgical interventions as demonstrated in this case often involve complex planning and decisions. Despite initial treatment of osteotomy and internal fixation for femur fracture with subsequent implant removal, progressive bowing of the tibia and femur necessitated further surgical correction. Patients with OI often develop new deformities over time due to underlying bone fragility and abnormal bone growth and this situation reflects the ongoing need for surgical interventions [6]. Osteotomies and using intramedullary rods are consistent with current practices aimed at correcting bony deformities and stabilizing in patients diagnosed with OI [7]. It is crucial to acknowledge that the use of high-speed instruments during osteotomies can negatively impact healing potential. High-speed tools may cause thermal necrosis and micro-damage to adjacent tissues, which can compromise the healing process. Therefore, the choice of instruments is vital in promoting optimal recovery. In this case, the decision to use a saw was based on its ability to provide a controlled cut while minimizing thermal injury. This consideration is particularly significant in patients with OI, where maintaining the integrity of surrounding soft tissues and vascular supply is essential for optimal healing. This comprehensive approach also reduces the risk of future fractures by providing structural support along with maintaining functional limb alignment.

In this particular case, a zoledronic acid infusion cycle was administered prior to the corrective surgery. Bisphosphonates, such as zoledronic acid, are crucial in the management of OI as these help in increasing bone density and reducing fracture risk [8]. Zoledronic acid use is also supported by various studies showing that bisphosphonates can significantly improve bone mineral density and reduce fracture rates in children with OI [9,10]. Bisphosphonate treatment prior to surgical intervention was aimed at enhancing bone strength and also to potentially improving surgical outcomes. The patient’s calcium and vitamin D levels were within a generally acceptable range. Vitamin D and calcium are essential for bone health and hence their levels need to be carefully monitored and managed in OI patients undergoing bisphosphonate therapy [9]. This patient’s calcium and vitamin D levels were adequate, which supports bone health and complements the effects of bisphosphonates treatment.

Postoperative care in these OI patients is critical as they require meticulous follow-up to monitor for complications and to ensure compliance with treatment protocols. The further management plan includes non-weight-bearing instructions and cast care [11]. Incorporating insights from mechanobiology can enhance the effectiveness of physical therapy in managing OI [12]. The emerging concept of mechanotherapy emphasizes the need to understand the cellular and biochemical responses to mechanical forces, which can inform rehabilitation strategies. By leveraging mechanotherapy approaches such as microdeformation, shockwave therapy, and distraction, osteogenesis clinicians may optimize recovery in OI patients, promoting improved bone regeneration and functional outcomes through targeted mechanical interventions. Regular follow-up and patient education on cast care and signs of complications are essential to prevent issues such as cast-related skin problems or signs of compartment syndrome [7].

## Conclusions

This case highlights the importance of a multimodal approach to OI management, integrating medication therapy with bisphosphonates, surgical correction, and careful postoperative care. This comprehensive strategy is important for improving outcomes and to improve quality of life. Further research and developments in pharmaceutical therapies and surgical approaches will improve the management associated with this challenging condition even further.
